# Comprehensive Model of Jumbo Squid *Dosidicus gigas* Trophic Ecology in the Northern Humboldt Current System

**DOI:** 10.1371/journal.pone.0085919

**Published:** 2014-01-20

**Authors:** Ana Alegre, Frédéric Ménard, Ricardo Tafur, Pepe Espinoza, Juan Argüelles, Víctor Maehara, Oswaldo Flores, Monique Simier, Arnaud Bertrand

**Affiliations:** 1 Instituto del Mar del Perú (IMARPE), Callao, Peru; 2 Institut de Recherche pour le Développement (IRD), UMR212 EME IFREMER/IRD/UM2, Sète, France; 3 Universidad Nacional Agraria La Molina, La Molina, Lima, Peru; Aristotle University of Thessaloniki, Greece

## Abstract

The jumbo squid *Dosidicus gigas* plays an important role in marine food webs both as predator and prey. We investigated the ontogenetic and spatiotemporal variability of the diet composition of jumbo squid in the northern Humboldt Current system. For that purpose we applied several statistical methods to an extensive dataset of 3,618 jumbo squid non empty stomachs collected off Peru from 2004 to 2011. A total of 55 prey taxa was identified that we aggregated into eleven groups. Our results evidenced a large variability in prey composition as already observed in other systems. However, our data do not support the hypothesis that jumbo squids select the most abundant or energetic taxon in a prey assemblage, neglecting the other available prey. Indeed, multinomial model predictions showed that stomach fullness increased with the number of prey taxa, while most stomachs with low contents contained one or two prey taxa only. Our results therefore question the common hypothesis that predators seek locally dense aggregations of monospecific prey. In addition *D. gigas* consumes very few anchovy *Engraulis ringens* in Peru, whereas a tremendous biomass of anchovy is potentially available. It seems that *D. gigas* cannot reach the oxygen unsaturated waters very close to the coast, where the bulk of anchovy occurs. Indeed, even if jumbo squid can forage in hypoxic deep waters during the day, surface normoxic waters are then required to recover its maintenance respiration (or energy?). Oxygen concentration could thus limit the co-occurrence of both species and then preclude predator-prey interactions. Finally we propose a conceptual model illustrating the opportunistic foraging behaviour of jumbo squid impacted by ontogenetic migration and potentially constrained by oxygen saturation in surface waters.

## Introduction

The ommastrephid jumbo squid *Dosidicus gigas* is the most abundant nektonic squid in the surface waters of the world ocean [1], [2] and supports the largest cephalopod fishery. This squid, endemic to the Eastern Tropical Pacific, is mainly distributed in the oceanic domain [3] over a wide bathymetric range [4]. *D. gigas* is a large squid with high fecundity [2], a rapid growth rate and a short life span (up to ∼32 months [5], [6]). The tolerance of this species to a wide range of environmental factors (temperature and oxygen) facilitates its geographic expansion [7]–[9], such as the recent invasion into California waters [4], [10].

*D. gigas* plays an important role in marine food webs both as predator and prey [11]. This abundant and voracious squid forages on a large variety of prey using prehensile arms and tentacles coupled with an efficient sensory system [12], [13]. The impact on exploited marine resources can be strong [4] and the broad trophic niche of jumbo squid is enhanced further by physiological abilities. This squid can undertake extensive vertical migrations, up to 1200 m, foraging on deep, mid-water and surface organisms [2], [7], [14], [15]. In addition, its presence within anoxic or hypoxic waters was validated by tagging experiments in the Californian Current System [15], [16]. Indeed, the eastern tropical Pacific is characterised by the presence of an oxygen minimum zone (OMZ) [17] and *D. gigas* is a part-time resident of the OMZ thanks to adapted behavior and specific metabolic characteristics [18], [19]. Jumbo squid vertical migrations impact the vertical energy flow, providing an efficient energy transport from the surface to deeper waters [7], [15].

Previous studies showed that the feeding ecology of jumbo squid is highly variable in time and space [20], [21]. The feeding ecology of jumbo squid was investigated in the eastern Pacific from stomach content [22]–[25] and stable isotopes [26]–[29]. By investigating stable isotope signatures along gladius, [28] showed that jumbo squids living in the same environment at a given time can have different historical backgrounds. These differences in life history strategies, illustrating a high plasticity, were confirmed by [29] who analysed carbon and nitrogen stable isotopes of individuals collected during 2008–2010. Here, we used an extensive dataset of more than 4000 stomachs sampled between 2004 and 2011 in the northern Humboldt Current to provide new insight on the size-related and spatiotemporal variability of feeding habits of *D. gigas*. We also decipher one paradox in the jumbo squid diet: why do they hardly forage on the tremendous biomass of anchovy *Engraulis ringens* distributed off coastal Peru? We show that the shallow OMZ in this area could hamper the co-occurrence of jumbo squids and anchovies, impacting jumbo squid foraging behaviour. We finally propose a conceptual model of jumbo squid trophic ecology including the ontogenetic cycle, oxygen conditions and prey availability.

## Materials and Methods

### Sample Collection

**Figure 1:**
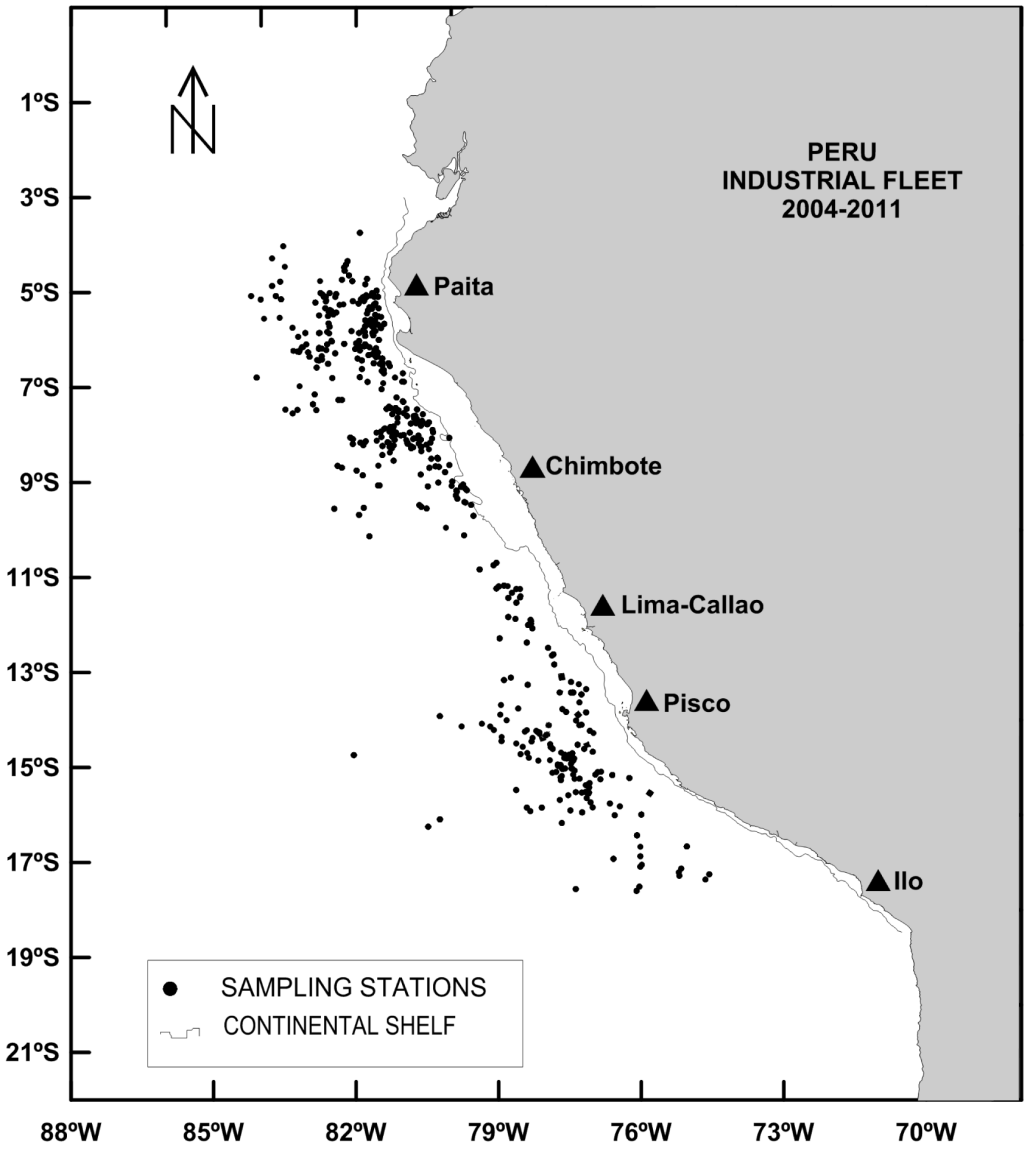
Location (black dots) of the sampling points of jumbo squids collected from the industrial jig fleet between 2004 and 2011.

A total of 5320 stomachs were collected from jumbo squids caught between 2004 and 2011 by the authorized industrial jigging fishery off Peru (3°S–17°S - from the coastal area to 605 km from the coast) (Fig. 1). No animals (squids i.e. invertebrates) were killed specifically for this research. Samples were collected by technicians of the Peruvian Sea Institute (IMARPE) aboard fishing vessels according to standard protocols. In each fishing set, 20 individuals were randomly sampled, covering the captured size range. On board or in the laboratory, length (mantle length ML, in cm) and total weight (in g) were measured and sex and maturity stages (I: immature; II: in maturing; III: mature; and IV: spawning) were determined according to [1], [29] and validated by [30]. Each fishing set was characterized according to the distance to the shelf break (negative to the continental shelf and positive towards offshore, in km), the season (austral summer, fall, winter and spring) and the diel period. Sea surface temperature anomalies (SSTA, in °C) were used as a proxy of environmental conditions.

### Stomach Content Analysis

All stomach contents were washed through a sieve mesh of 500 µm in order to retain prey remains and diagnostic hard parts (fish otoliths, cephalopod beaks, crustacean exoskeleton). Stomach contents were weighed and the different items constituting a single taxon were sorted, counted and weighed. Jigging vessels use 2 kW lights (no use of bait) to attract jumbo squids. Biases can be associated with fishing gear and tactic but jigging is recommended for diet studies [21]. Jigging avoids overestimating the occurrence of target commercial species in the stomach contents of jumbo squids that can feed after capture. Light is a powerful stimulus that attracts individuals independently of their satiety. In addition jumbo squids are known to be extremely voracious and thus can continue to feed once their stomachs are full. However, this fishing tactic and the squid voracity artificially increase the proportion of cannibalized jumbo squids in the stomach contents [31], [32]. To remove this unnatural feeding, the easily identifiable fresh jumbo squid portions were systematically eliminated from the stomach contents. Even after this procedure, jumbo squid was still by far the dominant prey by wet weight and reached 75%, indicating that fishery-induced cannibalism was not fully eliminated. This high rate was mainly due to 859 stomachs containing *D. gigas* only. We were therefore not able to precisely estimate the importance of natural cannibalism with our dataset that was still blurred by artificially induced cannibalized conspecifics. We thus removed these 859 stomachs and worked with the remaining 4461 (83.9%), from which 3618 were not empty (68% of the total number of stomachs) (Table 1). We probably eliminated some samples that were not affected by the fishing tactic but this protocol clearly allowed us to improve the relevance of the results.

**Table 1:** Overall description of sampled jumbo squid stomachs during 2004–2011.

	2004	2005	2006	2007	2008	2009	2010	2011	Total
N° Dietary groups	27	23	24	18	24	30	33	29	55
N° Stomachs	650	283	589	320	657	922	603	437	4461
N° Non-empty stomachs	520	224	479	239	542	740	523	351	3618
% Non-empty stomachs	80	79.2	81.3	74.7	82.5	80.3	86.7	80.3	81.1
Size range (cm)	21.0–104.5	28.7–91.0	27.4–98.0	28.3–109.5	14.3–112.5	23.6–111.5	16.8–108.6	24.5–114.2	14.3–114.2
Latitude range (°S)	5.0–15.5	4.7–15.2	5.7–15.2	4.3–10.7	5.1–17.6	3.7–16.0	4.8–17.6	4.0–16.2	3.7–17.6
Longitude range (°W)	75.8–82.3	76.6–82.6	76.3–81.9	79.2–83.8	74.6–83.0	76.0–84.0	75.0–82.8	77.0–84.2	74.6–84.2
Distance to the shelf break range (km)	−10.4–210	−15.4–245.8	5.7–218.6	15.5–260.2	23.5–254.5	16.1–342.4	20.6–553.9	62.1–330.8	−15.4–553.9
Distance to the coast range (km)	39.8–300.2	73.8–357.1	70.4–347.8	37.3–271.3	48.9–261.4	40.3–390.6	40.9–604.7	77.3–363.8	37.3–604.7

Identifiable fresh remains and diagnostic hard parts were used to determine the number of each prey item. For fish otoliths and cephalopod beaks, the maximum number of left or right otoliths and the greatest number of either upper or lower beaks were used to estimate the number of fish and cephalopods, respectively. Prey items were identified to the most precise possible taxonomic level using keys and descriptions for fish [33], [34], crustaceans [35], [36] cephalopods [37], and other molluscs [38]. The degree of digestion of the stomach contents can preclude the identification of all prey remains. However, fresh remains made up the largest percentage of our stomach content samples. The meticulous analyses of the stomach contents performed in our laboratory allowed us to divide into broad prey classes (Cephalopods n/i, Teleosteii n/i, Crustacea n/i) the unidentified remains (see Table S1). A total of 55 prey taxa were identified at different taxonomic levels (see Tables S1 and S2). Prey were quantified by frequency of occurrence, numbers and wet weight. Mean percentages by number (%N) and by weight (%W) were computed by averaging the percentages of each prey taxon found in the individual stomachs. We thus treated individual squid as the sampling unit, allowing us to compute standard deviations [39]. As the identification level was not homogeneous during the 2004–2011 period, we aggregated prey in eleven groups based on their consistency and their ecological importance in the Humboldt Current system (Table S1).

A stomach fullness weight index (FWI, in %) was calculated [40]:
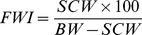
where SCW is the wet weight of the stomach content (g) and BW the body wet weight of the individual (g).

### Data Analyses

A clear relationship exists between squid size and maturity stages (Fig. S1) indicating that size is, to some extent, a proxy for ontogenetic processes. Therefore we used size to investigate life cycle effect on jumbo squid diet. Jumbo squid diet did not significantly vary with sex (results not shown). This factor was thus not taken into account in further analyses. Jumbo squid were generally captured by jigging after dusk and therefore night samples (62%) dominated the dataset. Preliminary analyses were performed on night data and on the whole data set. Results were similar and we therefore reported results with the complete set of data only.

In order to analyse the potential effects of explanatory variables on the number of taxa per stomach, a proportional-odds model for ordinal response [41] was fitted to the vector of prey richness, i.e. the number of different taxa recovered in each stomach *(y_i_)_i≥1_* that was assumed to be a realization of a random variable *Y*. *Y* takes its values in the set *E = {1, 2, …, S}* with *S* equals the maximum observed richness in the 3618 non empty stomachs. The model was written in terms of the cumulative probability function of *Y*, conditional on three continuous exogenous covariates (size, stomach fullness index and distance to the shelf break). The logistic form was chosen to predict the probabilities of observing different prey richness as a function of the covariates of interest.

The potential effects of explanatory variables (mantle length, season, distance to the shelf break, SSTA) on stomach fullness index and diet of jumbo squid were first investigated using Kruskal-Wallis (KW) non-parametric tests. This preliminary approach allowed us to perform an initial inspection of the dataset. Length, distance to the shelf break and SSTA were then each divided in four ordered categories, according to their ecological interpretation (the number of stomachs is given for each category); for mantle length: less than 40 cm (559), 41–60 cm (1553), 61–80 cm (934), over 80 cm (572); for distance to the shelf: less than 50 km (840), 51–75 km (682), 76–130 km (829), over 130 km (1267); for SSTA: less than −1.5°C (616), −1.49 to −0.5°C (899), −0.49 to 0.5°C (1299), over 0.5°C (804). Stomach numbers for the four seasons were: summer (690 stomachs), fall (1068), winter (997) and spring (863). However this approach did not account for dependence and interactions between explanatory variables, and then did not elucidate the complex relationships between the type of prey and the environmental factors. In addition the sampling scheme was very unbalanced in space and time. To cope with these issues, we ran a classification and regression tree (CART) analysis proposed by [42] and adapted to diet data by [43]. Classification tree was used here as a tool to identify the relationships between explanatory variables and the distribution of prey groupings. This non-parametric method gives a clear picture of the structure of the data, and allows an intuitive interpretation of the interactions between variables. The classification tree uses a partitioning algorithm to estimate a series of binary decision rules that divide the data into smaller homogeneous subgroups in an optimal way. The whole dataset is represented by a single node at the top of the tree. Then the tree is built by repeatedly splitting the data. Each split is defined by a simple rule based on a single explanatory variable. Splits are chosen to maximize the homogeneity of the resulting two nodes. We followed the approach of [43] and transformed the diet data as follows: each row represents a unique predator-prey combination, where the proportion by wet weight of one of the eleven prey taxa potentially present in the stomach of a predator is used as a case weight for the classification tree. As the splitting procedure grows an overlarge tree, we applied a prune back procedure to keep the tree reasonably small to focus on the first most informative splits. Each terminal node (or leaf) of the final tree is characterized by a predicted prey distribution (percentage by weight of 11 groups), given three explanatory continuous variables (stomach fullness index, distance to the shelf break and SSTA) and two categorical variables (season: summer, fall, winter and spring; and individual size (cm) divided into four ordered categories). Year effect was also tested but this factor had no significant effect on the pruned tree and was removed from the final model (Table S2 for detailed data per year).

Analyses were conducted using the statistical open source R software (R Core Team 2013), with the *MASS* package for the proportional odds-model [44] and the *rpart* package for the classification tree.

## Results

### Overall Diet Description

**Figure 2:**
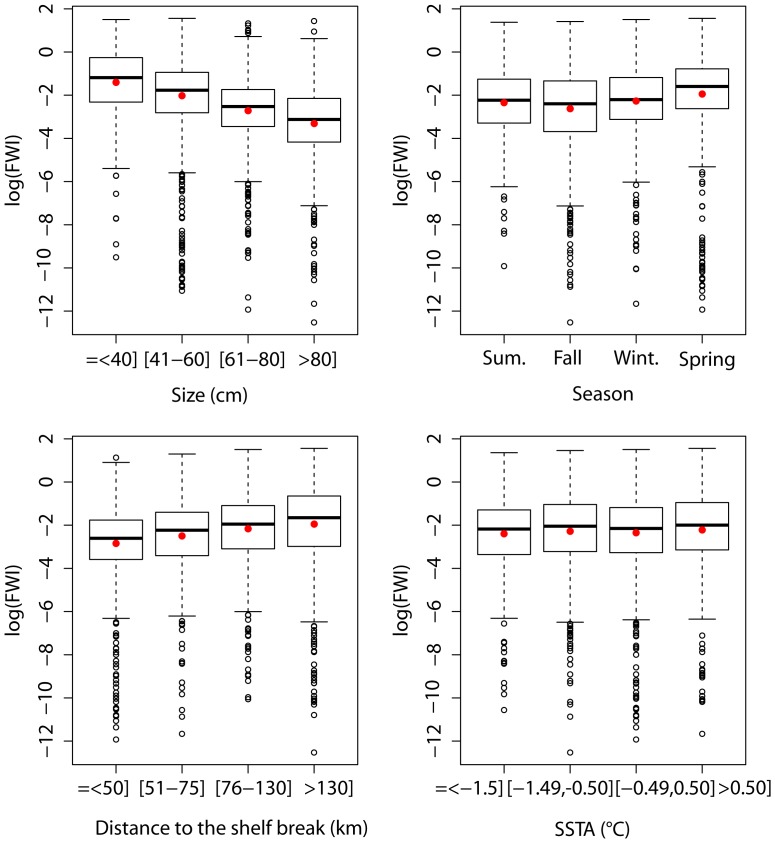
Distribution of the logarithm of the Fullness Weight Index (log(FWI)) according to the individual size (A), the season (B), the distance to the shelf-break (C), and the Sea Surface Temperature Anomaly (SSTA) (D).

The size of the 4461 selected squids ranged from 14.3 to 114.2 cm ML (Table 1). Overall, 19% of the stomachs were empty. For the 3618 non-empty ones, stomach fullness weight index (see Fig. S2 for details on FWI distribution) decreased significantly with size (Fig. 2A; KW, H = 499.6, df = 3, *P*<0.01) and increased significantly with distance to the shelf (Fig. 2C; KW, H = 177.8, df = 3, *P*<0.01). On the opposite, effect of SSTA was not significant (Fig. 2D; KW, H = 8.5, df = 3, *P*>0.05), but slightly higher values of stomach fullness weight index occurred in spring (Fig. 2B; KW, H = 93.8, df = 3, *P*<0.01).

Cephalopoda (*Dosidicus gigas* and other Cephalopoda) were the dominant food source in %O, %N and %W (Table 2). Both taxa were observed in 13.2 and 44% of the stomachs, respectively, and contributed together 40% by weight and 30% by number. The Phosichthyidae *Vinciguerria lucetia* occurred frequently in the stomach contents (36%), representing an average percentage of nearly 20% by weight and 25% by number. The three Myctophidae taxa (*Myctophum* spp., *Lampanyctus* sp. and other Myctophidae) occurred in 1577 samples (8.4, 13.6 and 21.7% respectively), and contributed 15% by weight and 18.3% by number. Teleosteii were frequent in the stomachs (21.7%) and represented 12.7% by weight and 11.7% by number.

**Table 2:** Distribution of the eleven dietary groups recovered from jumbo squid stomach contents off Peru between 2004 and 2011.

Dietary groups	Prey code	N° Stomachs	%FO	%W	%N
*Dosidicus gigas*	Dgig	478	13.2	8.6 (±25.5)	3.4(±11.7)
Other Cephalopoda	Ceph	1591	44.0	31.2 (±44.2)	26.4(±39.7)
Euphausiidae	Euph	299	8.3	6.4 (±23.7)	7.8(±26.3)
*Pleuroncodes monodon*	Pleu	83	2.3	1.7 (±12.4)	1.7(±12.5)
Engraulidae	Engr	142	3.9	2.7 (±15.3)	2.1(±12.4)
*Lampanyctus* sp.	Lamp	491	13.6	4.6 (±19.6)	5.1(±17.8)
*Myctophum* spp.	Mycg	302	8.4	3.6 (±17.5)	3.5(±15.4)
Other Myctophidae	Mycf	784	21.7	6.7 (±23.7)	9.7(±24.4)
*Vinciguerria* *lucetia*	Vluc	1299	35.9	19.7 (±37.6)	24.4(±37.8)
Teleosteii	Tele	786	21.7	12.7 (±31.7)	11.7(±28.2)
Other	Othe	333	9.2	2.0 (±12.8)	8.8(±17.1)

For each prey group are indicated, the corresponding number of stomachs (N° Stomachs), the frequency of occurrence (%FO), and the percentage of prey group per stomach by weight (%W) and by number (%N) (mean value ± standard deviation).

**Figure 3:**
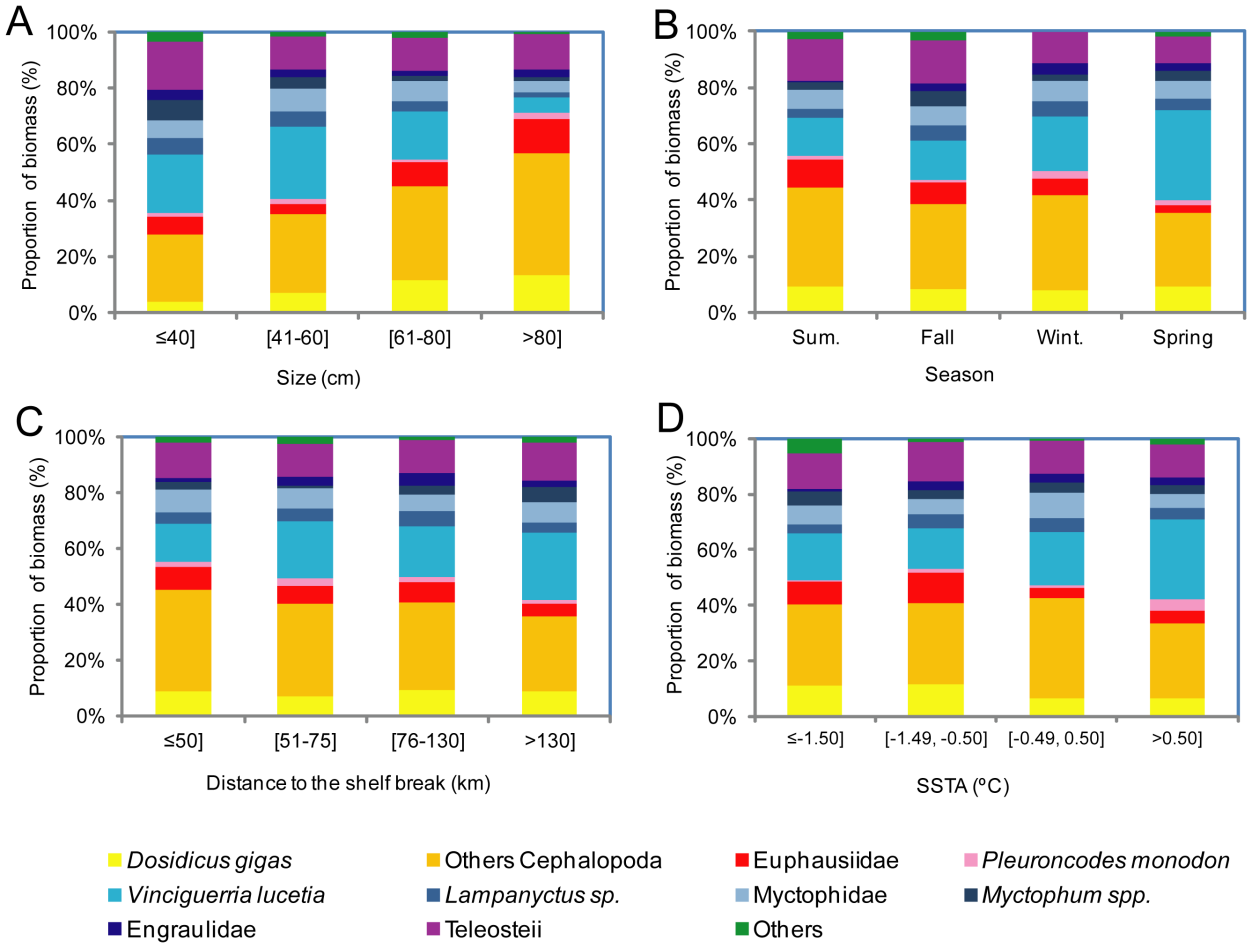
Jumbo squid diet composition in weight (%) according to the individual size (A), the season (B), the distance to the shelf-break (C), and the Sea Surface Temperature Anomaly (SSTA) (D).

The diet composition of jumbo squid in weight varied according to size (Fig. 3A). The main pattern was the steady increase of the percentage of cephalopods with size: *D. gigas* and other Cephalopoda accounted for 24.3% of the diet of small squids (ML<40 cm) and reached 43.2% for large squids with ML>80 cm. The percentage of Euphausiidae also increased significantly (Table S3) with size, except for the smallest squids: 6% for the size class under 40 cm, 3.5% in individuals between 40 and 60 cm, 8.4% in individuals between 60 and 80 cm, and 12.4% in individuals larger than 80 cm. On the opposite, the importance of *V. lucetia* (21% to 5.6%) and *Myctophum* sp. (7.2% to 1.3%) decreased significantly while jumbo squid increased in size (Table S3).

No clear tendency appeared with the season (Fig. 3B), except a significantly higher percentage of *V. lucetia* (32%) in spring and less Cephalopoda (26%), Euphausiidae (2.8%) and Teleosteii (9.3%) (Table S3). In summer, Euphausiidae were at their maximum (10%) while the percentage of *V. lucetia* was low (13.7%) and Engraulidae were very rare (0.4%).

The diet composition of *D. gigas* varied significantly with the distance to the shelf break (Fig. 3C; Table S3): Euphausiidae slightly decreased, Cephalopoda decreased from 36.3% inside the 50 km to 26.8% out of the 130 km, while percentages of *V. lucetia* increased from 13.8% inside the 50 km to 24.2% out of the 130 km. The percentage of Engraulidae also increased with the distance to the shelf break except for distances greater than 130 km.

Diet changed according to SSTA (Fig 3D). Trend from negative towards positive anomaly was associated to a significant increase in *V. lucetia* (from ∼15 to 28.6%) and a significant decrease in cannibalism (from ∼11 to 6.6%) (Table S3).

### Prey Taxa Richness

Based on the detailed 55 prey taxa, the prey richness in the stomachs was very low. A maximum of seven prey taxa was observed in one stomach only, while a single prey taxon was recovered in 48.0% of the stomachs and 30.7% had two prey taxa (mean = 1.87, sd = 1.10). Results were similar with the eleven aggregated taxa: a maximum of seven prey taxa, 48.6% with one prey taxon and 31.1% with two prey taxa (mean = 1.82, sd = 1.02). Consequently, analyses were performed with the eleven aggregated taxa (Table 2).

**Figure 4:**
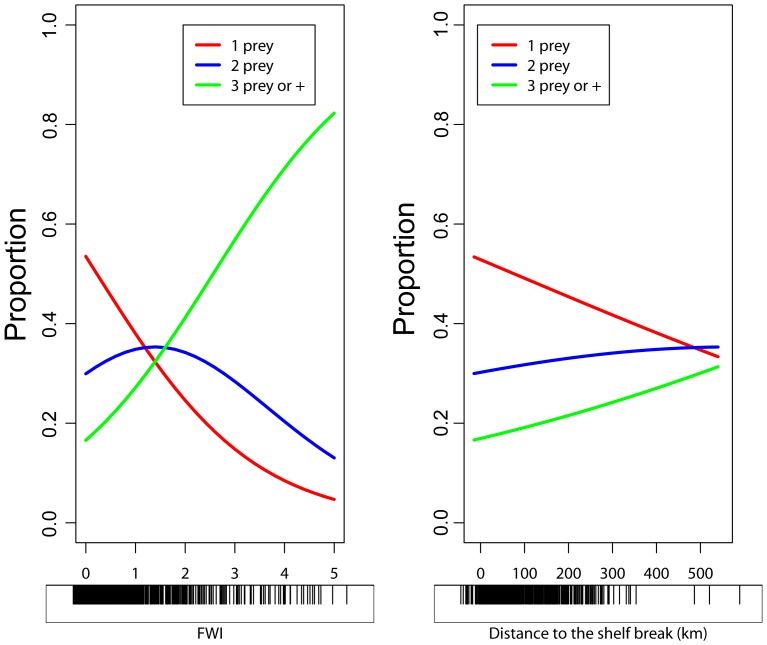
Proportional odds model. Prediction of the number of prey groups (1, 2, 3 or more) in a given stomach according to the fullness weight index (FWI) (**A**) and the distance to the shelf-break (**B**). Black tick marks under the x-axes show the location of the data points.

According to the Akaike information criterion (AIC), the proportional-odds model with two covariates (fullness and distance to the shelf, AIC = 8691) was the most parsimonious (adding squid size did not improve the fit, AIC = 8692). The estimated values of the parameters were used to compute the probabilities of observing 1, 2, or 3+ (i.e., at least 3) prey taxa in a stomach as a function of stomach fullness or distance to the shelf. Increasing the stomach fullness led to a sharp increase in the probability of recovering 3+ prey taxa in a stomach and to a marked decrease of the probability to observe only one taxon (Fig. 4A). After a short plateau, the probability for two taxa roughly decreased with stomach fullness too. On the other hand, the probability to find one taxon only decreased with the distance to the shelf, while the probabilities to recover more than two prey taxa increased with this covariate (Fig. 4B).

### Multivariate Approach

**Figure 5:**
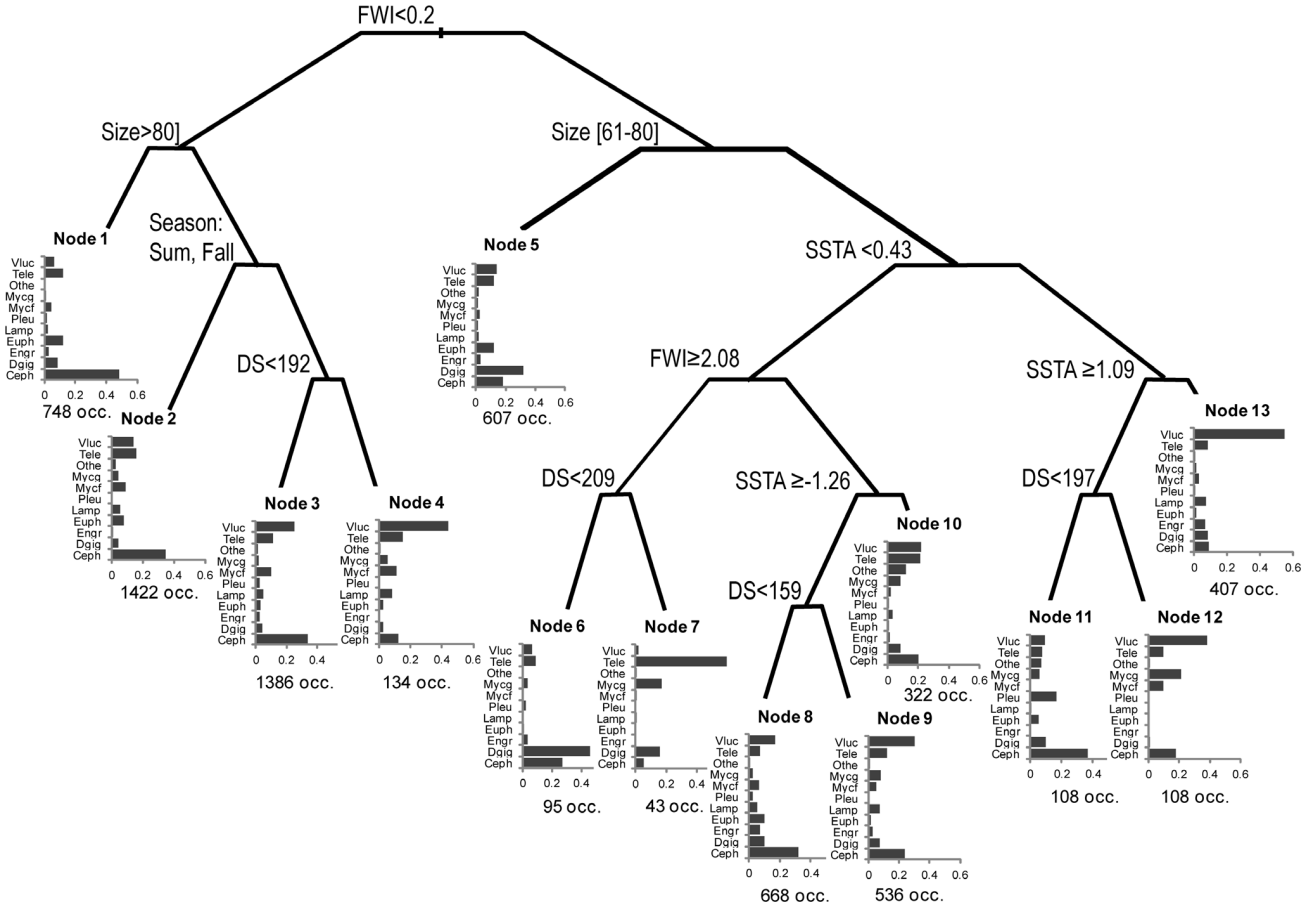
Classification tree of jumbo squid diet (prey groups) according to the Fullness Weight Index (FWI), the Distance to the Shelf (in km) (DS), the Sea Surface Temperature Anomaly (SSTA, in °C), the mantle length (Size in cm) and the Season. For each final node, the predicted probabilities of occurrence of the 11 prey groups is detailed (histograms) and the number of prey occurrences (occ) is given. See Table 2 for prey codes.

The pruned classification tree showed 13 nodes (Fig. 5A). The first split separated four nodes corresponding to a very low fullness (<0.2) from the others. Among this group, the nodes 1 to 3 predicted diet compositions dominated by cephalopods (predicted cephalopod probability = 0.48, 0.35 and 0.34, respectively), which occurred more likely in individuals larger than 80 cm ML (node 1), in individuals smaller than 80 cm ML caught in summer and fall (node 2), and in individuals located within the 191 km from the shelf break caught during winter and spring (node 3). The node 4 however showed a high incidence of *V. lucetia* (predicted probability = 0.44) at a distance to the shelf break higher than 191 km, in winter and spring. The node 5 showed a high probability of cannibalism (predicted probability = 0.32) for medium size (between 60 and 80 cm ML) individuals with stomach fullness higher than 0.2. From the node 6 on, squids had a smaller ML (less than 60 cm). The node 6 also showed a high probability of cannibalism (predicted probability = 0.46) for SSTA <0.425°C, in individuals with fullness greater than 2.08, located at less than 209 km to the shelf break. The node 7, characterised by the teleosteii (predicted probability = 0.60), had the same characteristics than the node 6, except a more offshore location. Nodes 8 to 10 showed a relatively balanced diet and were separated from nodes 6 and 7 by a lower fullness (<2.08). Nodes 11 to 13 corresponded to fullness ≥0.2, size <60 cm and SSTA ≥0.425°C. Node 11 was associated to high SSTA (≥1.09°C), short distance to the shelf break (<197 km), and predicted a dominance of cephalopods (predicted probability = 0.37). In nodes 12 (distance to the shelf break greater than 197 km) and 13 (SSTA<1.09°C), *V. lucetia* was largely dominant (predicted probability = 0.38 and 0.55, respectively).

## Discussion

This work is based on an extensive dataset on jumbo squid diet encompassing a large range of spatiotemporal location and sizes. Beyond the usual diet description, our results allowed us to provide new knowledge on jumbo squid trophic ecology, in particular on prey distribution under different environmental conditions and on the role that could be played by the dissolved oxygen.

### Prey Richness

Using the detailed (55 taxa) or aggregated (eleven taxa) databases, prey richness in stomachs was similar with an average of 1.8 taxa per stomach. This unexpected result has several consequences. It first empirically validates the eleven aggregated taxonomic groups (Table S1). Second, it shows that when jumbo squid foraged on one prey among the 55 taxa, it did not feed on extra prey belonging to the same assemblage among the eleven aggregated taxa. A spatial segregation of prey of jumbo squids may explain this observation. If a taxon from one group of the eleven aggregated taxa occurred in a location where jumbo squids seek their prey, the probability of the presence of an extra taxon belonging to the same group may have been low. On the contrary, jumbo squid could select the most abundant or energetic taxon of a group, neglecting the other available prey belonging to the same group. Our data did not support either of these hypotheses. However, Predictions of the multinomial model showed that stomach fullness increased with the number of prey taxa, while most of the stomachs with low contents contained one or two prey taxa only. We could have expected an opposite pattern. Indeed, top predators such as tuna exhibit high foraging efficiency (high fullness) in presence of large and dense monospecific prey aggregations in surface layers (e.g., [45]–[47]). Once a prey concentration of one target species is detected, tunas can feed on this concentration until satiation [48]. On the contrary, when prey are scarce and dispersed in the environment [49], tunas forage on a higher diversity of prey but with a lesser efficiency [50]. For jumbo squid our results therefore question the usual hypothesis that top predators may seek locally dense aggregations of monospecific prey.

### Dietary Composition, Environmental Conditions and Size-related Patterns

Identifying cephalopods food is tricky [11]: the beak can bite off small pieces of tissue of large prey; diagnostic hard parts of prey, such as fish otoliths, skeletons, crustacean integuments or cephalopod beaks are often rejected. Selective rejection can also occur and blur diet composition. In addition, digestion is known to be rapid among cephalopods. However, we carefully dealt with the intrinsic biases linked to the data sampling and with the identification of prey items that was carried out by the same scientific team following a constant protocol. Consequently, the extensive set of data over a large time period allowed us to elucidate the foraging behaviour of jumbo squids in the northern Humboldt Current system. We assume that changes in prey composition according to squid size and spatiotemporal features were more related to prey accessibility rather than to specific/size-related preferences. Jumbo squid perform ontogenetic migration with small individuals distributed further offshore than larger individuals [5]. Spawning in less productive offshore waters is used by other species to avoid predation on first stages (e.g. the South Pacific jack mackerel, *Trachurus murphyi*; [51]). This spatial dynamics is here also evidenced with small individuals distributed further offshore than the large ones. However the biggest ones (>800 cm ML) seem to move back offshore, probably to spawn [52] but not as far as the smallest individuals that are advected further offshore at early stages. Note that warmer waters (offshore in our case) are suitable for spawning [25]. Prey composition in the stomach contents matched this pattern. Euphausiids contributed at a higher level as prey of large rather than of small squids, according to the known spatial distribution of euphausiids. Ballón et al. [53] showed indeed that the biomass of euphausiids was maximal off the shelf-break until a distance of ca. 150 km, an area where the larger individuals spawn [52]. Therefore, contrary to most past studies [1], [20], [29], [32], [54] zooplankton contribution does not systematically decrease with the size. In addition, isotope signatures along jumbo squid gladius in the northern Humboldt Current system showed that large individuals can substantially forage on low trophic levels [28].

Mesopelagic fish (*V. lucetia* and myctophiids) recovered in the jumbo squid stomachs confirmed the structuring role of spatial matching in the jumbo squid-prey interactions. This prey group contributed mainly during spring and far from the coast, when jumbo squid was more offshore. In addition, small jumbo squids distributed far from the coast consumed more mesopelagic fish than larger individuals located closer to the coast. This pattern was unexpected again, but is in accordance with the distribution pattern of mesopelagic fish that are distributed more offshore than euphausiids [55].

Cannibalism contributed greater than 8% by weight. High levels of cannibalism were frequently observed in jumbo squid [20], [21]. Yet, cannibalism can be overestimated depending of the fishing gear used for capture [21], [56]. In this study we followed various steps to remove as far as possible artificially induced cannibalism. On the other hand, cannibalism may also be underestimated. Indeed, squid muscles sections with a high degree of digestion are difficult to determine. When it was not possible to identify the squid prey species, the corresponding items were incorporated in the group of other cephalopods. Thus some digested *D. gigas* were most likely classified as ‘other cephalopoda’.

Several hypotheses are proposed to explain cannibalism in squid. This behavior may be part of an energy storage strategy of the population, allowing cephalopod to react to favorable and adverse environmental conditions by increasing or reducing their number [56]. Cannibalism can also provide a competitive advantage among young and adults and can be beneficial for survival during periods of food shortage [57]. We observed the classic pattern of steady increase of cannibalism with size related to the increase in predator’s ability to capture and handle the prey [58], [59]. Large specimens can access to highly energetic food when feeding on conspecifics [60]. However, the relative spatial segregation of this species by size [5] may be a response to limit cannibalism on juveniles.

### The Anchovy Paradox: Does Oxygen Matter?

**Figure 6:**
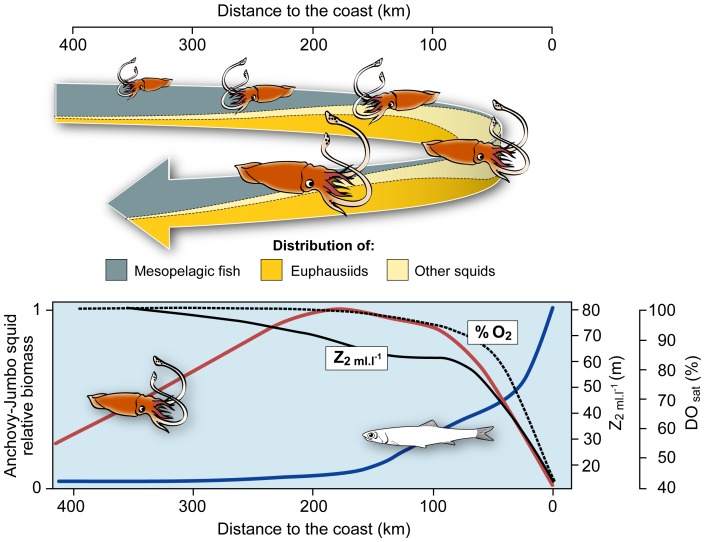
Conceptual model and cross-shore profiles of oxygen and organisms distribution. The lower panel shows the mean (spline smooth) cross-shore profiles of dissolved oxygen saturation in % (grey dashed line), depth of the 2 ml.l^−1^ isoline in m (black solid line) and the acoustic-estimated biomass of anchovy (blue solid line) and the jumbo squid acoustic-estimated biomass (red solid line). Oxygen and anchovy data come from Bertrand et al. (2011); jumbo squid data come from IMARPE, unpublished data. Note that the oxygen data cover the range 7°S to 18°S. The upper part shows the cross-shore distribution of jumbo squid along its ontogenetic cycle. The colours in the arrow represent the schematic range of distribution and proportional abundance of the three main prey groups i.e., the other cephalopoda, euphausiids and mesopelagic fish.

In the California Current system *D. gigas* forages substantially on coastal fish, particularly anchovy (*Engraulis mordax*) [25], [61]. Surprisingly *D. gigas* consumes very few anchovy in Peru, whereas a tremendous biomass of anchovy is potentially available. Furthermore, off Peru, anchovy is concentrated in schools or dense aggregations within the thin surface oxygenated layer [62], [63], which makes anchovy an easy prey for mobile predators [64]. Unlike in California [25], the jumbo squid distribution hardly overlaps with that of anchovy, which is very coastal (Fig. 6). Why does jumbo squid not distribute closer to the coast and benefits from the huge anchovy stock? Oxygen may be the answer. Anchovy is not adapted to anoxia and cannot enter the oxygen minimum zone. However this small fish (oxygen supply per body size decreases as fish size/weight increases) can forage at low cost (so low oxygen demand) on macrozooplankton and is thus adapted to inhabit the unsaturated surface coastal waters [65]. On the contrary, jumbo squid is adapted to anoxia since it undertakes diel vertical migration and occupies the oxygen minimum zone (OMZ) during the day [15], [16], [18], [66]–[69]. *D. gigas* succeeds in the OMZ by managing hypoxia *via* metabolic suppression [18], [19], [66], [67], coupled with a high-affinity respiratory protein, the hemocyanin [69]. However normoxic conditions are needed in surface during the night to supply the oxygen demand that was not achieved in hypoxic waters at greater depths [69], [70]. In coastal Peru the OMZ is much more intense than in California, the upper OMZ is shallower and, above the oxycline, oxygen concentration and saturation are low [66]. In such conditions jumbo squid may be prevented to enter the coastal waters where the anchovy is situated, as was previously evidenced for sardine [65]. Indeed, off Peru, the abundance of jumbo squid biomass increases with oxygen saturation (Fig. 6). When upwelling is strong, anchovy partly distributes off the shelf break and should be more accessible to jumbo squid. However, such conditions correspond also to an extension of the surface oxygen unsaturated waters [65].

### Synthesis

As a synthesis we propose a comprehensive model of jumbo squid *Dosidicus gigas* trophic ecology in the northern Humboldt Current system (Fig. 6). Small jumbo squid (<400 mm) are mostly distributed far offshore where they largely forage on mesopelagic fish. As they grow, they move closer to the coast and increase their consumption of other cephalopoda. However, off Peru, contrarily to other systems [25], *D. gigas* does not occupy very coastal waters where a huge biomass of anchovy is present. We hypothesize that jumbo squid cannot enter the coastal waters that present low surface oxygen saturation. Although jumbo squid can forage in hypoxic deep waters it needs surface normoxic waters afterwards [69]. Oxygen concentration may thus limit the co-occurrence of both species and then preclude predator-prey interactions. Large squids move further offshore (without reaching the oceanic distribution of smaller jumbo squids), and increase their consumption of squids (including jumbo squid) and euphausiids. Note that euphausiids consumption is rather low considering its availability, indicating that *D. gigas* may seek out more energetic prey. The global pattern we described illustrates the opportunistic foraging behaviour of jumbo squid, which is impacted by ontogenetic migration and most likely by oxygen conditions. Also, even if the global scheme described in Figure 6 seems consistent [28], [71], high variability exists between individuals and the differences in jumbo squid life history strategies highlight the high degree of plasticity of the jumbo squid and its high potential to adapt to environmental changes.

## Supporting Information

Figure S1 Distribution frequency of jumbo squid maturity stages (I: immature; II: in maturing; III: mature; and IV: spawning) according to mantle size.
(TIF)

Figure S2 Distribution of the fullness weight index (FWI) of non-empty jumbo squid stomach.
(TIF)

Table S1 Overall description of the 55 prey taxa observed in jumbo squid stomach sampled off Peru during 2004–2011. Are indicated, the taxonomic information, the mean value (±standard deviation) of the proportion by weight (%Weight) and by number (%Number) as well as the frequency of occurrence (%Occurrence).
(DOCX)

Table S2 Yearly description of the 55 prey taxa observed in jumbo squid stomach sampled off Peru during 2004–2011. Proportion by weight: %W; proportion by number: %N; frequency of occurrence: %O.
(DOCX)

Table S3 Result of the Kruskal-Wallis test performed on the 11 dietary groups according to Size, Distance to the shelf-break, Season and SSTA. Significant differences are in bold.
(DOCX)
