# Supplementary figures and images for: Comprehensive Model of Jumbo Squid Dosidicus gigas Trophic Ecology in the Northern Humboldt Current System
Source: PLoS One. 2014 Jan 20;9(1):e85919. doi: 10.1371/journal.pone.0085919 (PMC3896428; doi:10.1371/journal.pone.0085919)

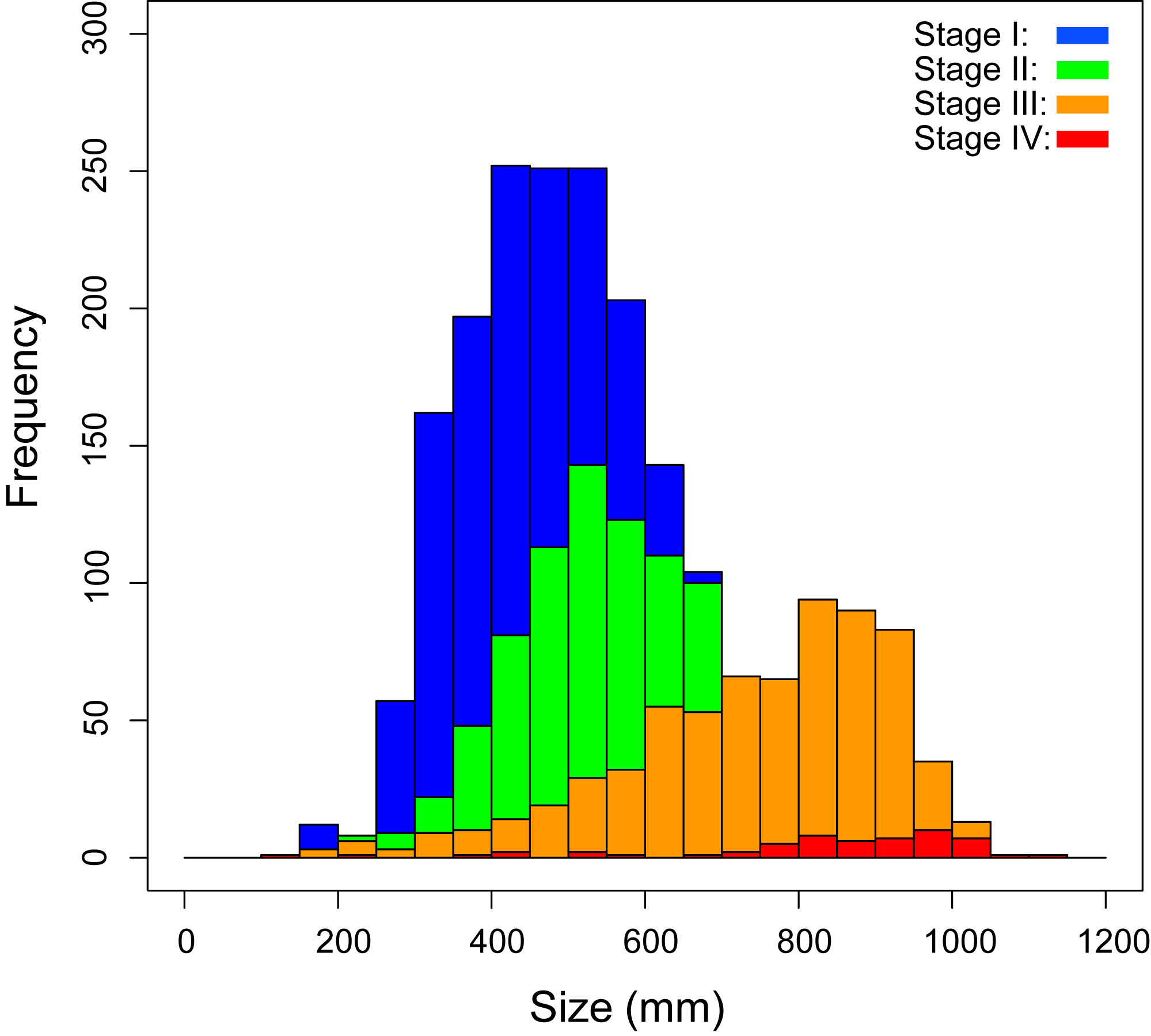

Supplement: Figure S1 — Distribution frequency of jumbo squid maturity stages (I: immature; II: in maturing; III: mature; and IV: spawning) according to mantle size. (TIF) [file pone.0085919.s001.tif]

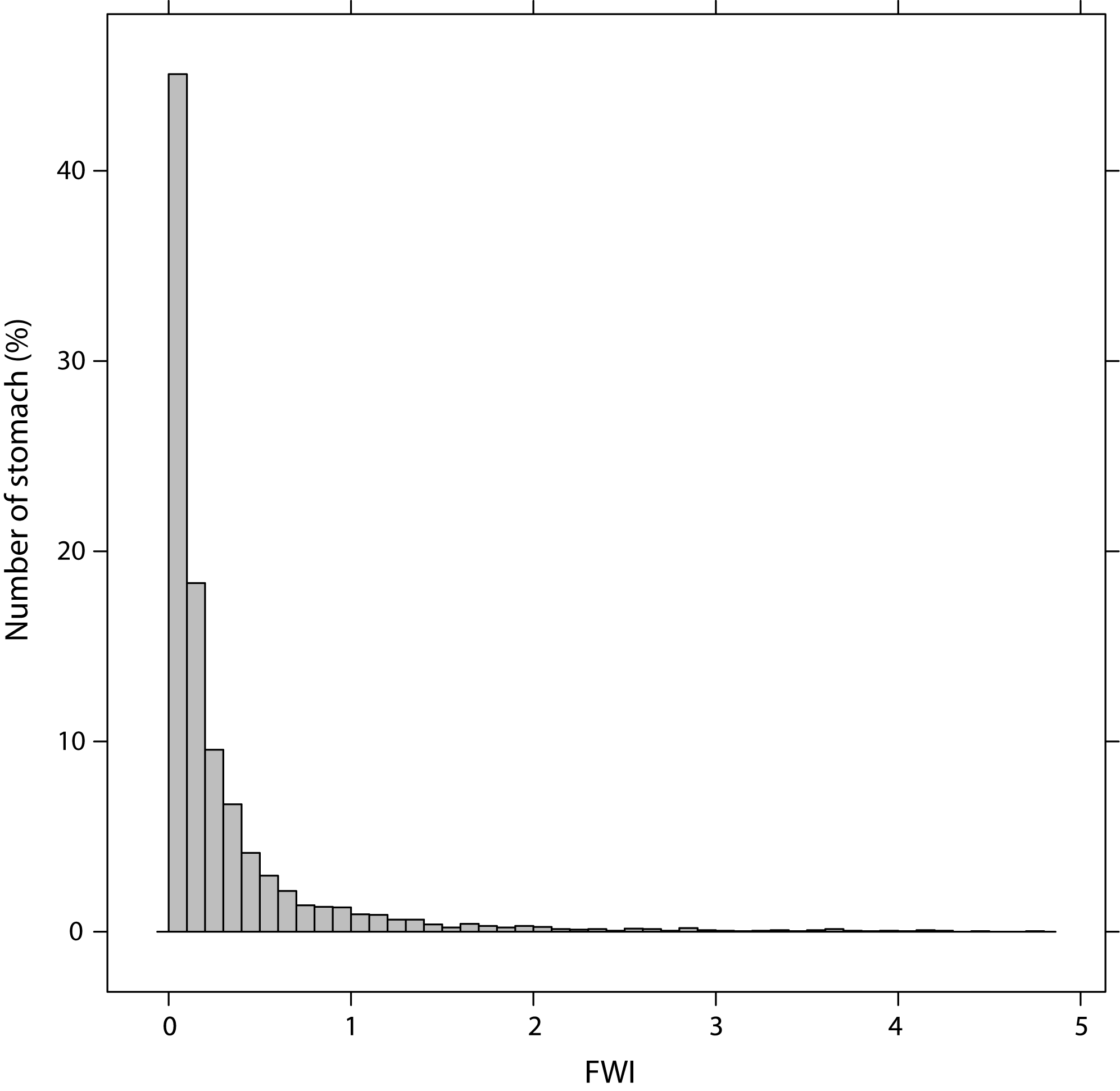

Supplement: Figure S2 — Distribution of the fullness weight index (FWI) of non-empty jumbo squid stomach. (TIF) [file pone.0085919.s002.tif]
