# Supplementary material for: Comprehensive Model of Jumbo Squid Dosidicus gigas Trophic Ecology in the Northern Humboldt Current System
Source: PLoS One. 2014 Jan 20;9(1):e85919. doi: 10.1371/journal.pone.0085919 (PMC3896428; doi:10.1371/journal.pone.0085919)
Supplement: Table S1 — Overall description of the 55 prey taxa observed in jumbo squid stomach sampled off Peru during 2004–2011. Are indicated, the taxonomic information, the mean value (±standard deviation) of the proportion by weight (%Weight) and by number (%Number) as well as the frequency of occurrence (%Occurrence). (DOCX) [file pone.0085919.s003.docx]

Table S1. Overall description of the 55 prey taxa observed in jumbo squid stomach sampled off Peru during 2004-2011. Are indicated, the taxonomic information, the mean value (±standard deviation) of the proportion by weight (%Weight) and by number (%Number) as well as the frequency of occurrence (%Occurrence).

| **Phylum** | **Class** | **Order** | **Family** | **Species** | **Dietary groups** | **% Weight** | **% Number** | **% Occurrence** |
| --- | --- | --- | --- | --- | --- | --- | --- | --- |
| Mollusca | Gastropoda | Thecosomata | Cavoliniidae | *Cavolinia uncinata* | Other | 0.07 (± 2.16) | 0.19 (± 3.49) | 0.44 |
|  |  |  |  | *Diacria* spp. | Other | 1.03 (± 9.13) | 2.39 (± 13.17) | 4.56 |
|  |  |  | Thecosomata n/i |  | Other | 0.00 (± 0.01) | 0.03 (± 1.02) | 0.08 |
|  |  | Littorinimorpha | Atlantidae | *Atlanta* spp. | Other | 0.00 (± 0.02) | 0.11 (± 1.38) | 1.08 |
|  |  |  | Naticidae | *Natica* spp. | Other | 0.01 (± 0.83) | 0.18 (± 2.37) | 1.44 |
|  |  | Gastropoda n/i |  |  | Other | 0.00 (± 0.05) | 0.03 (± 0.91) | 0.19 |
|  | Bivalvia | Solemyoida larvae |  |  | Other | 0.00 (± 0.00) | 0.01 (± 0.36) | 0.17 |
|  | Cephalopoda | Octopoda | Argonautidae | *Argonauta* sp. | Other Cephalopoda | 1.21 (± 10.25) | 1.06 (± 8.41) | 2.65 |
|  |  | Myopsida | Loliginidae |  | Other Cephalopoda | 0.21 (± 4.47) | 0.21 (± 4.45) | 0.25 |
|  |  | Oegopsida | Enoploteuthidae | *Abraliopsis affinis* | Other Cephalopoda | 3.65 (± 17.51) | 3.19 (± 15.49) | 6.63 |
|  |  |  | Ommastrephidae | *Dosidicus gigas* | *Dosidicus gigas* | 8.57 (± 25.49) | 3.41 (± 11.69) | 13.21 |
|  |  | Paralarve of Cephalopoda |  |  | Other Cephalopoda | 0.00 (± 0.01) | 0.00 (± 0.12) | 0.03 |
|  |  | Eggs of Cephalopoda |  |  | Other Cephalopoda | 0.03 (± 1.66) | 0.03 (± 1.66) | 0.03 |
|  |  | Cephalopods n/i |  |  | Other Cephalopoda | 26.11 (± 42.21) | 21.95 (± 37.72) | 35.74 |
| Arthropoda | Crustacea n/i |  |  |  | Other | 0.37 (± 5.67) | 0.44 (± 5.87) | 0.72 |
|  | Maxillopoda | Calanoida | Aetididae | *Aetideus* sp. | Other | 0.00 (± 0.00) | 0.00 (± 0.18) | 0.03 |
|  |  |  | Oncaeidae | *Oncaea* sp. | Other | 0.00 (± 0.00) | 0.02 (± 0.81) | 0.11 |
|  |  |  | Calanoida n/i |  | Other | 0.00 (± 0.06) | 0.02 (± 0.99) | 0.03 |
|  | Ostracoda |  |  |  | Other | 0.01 (± 0.45) | 0.03 (± 1.50) | 0.08 |
|  | Malacostraca | Amphipoda | Gammaridea |  | Other | 0.06 (± 2.33) | 0.08 (± 2.63) | 0.11 |
|  |  |  | Amphipoda n/i |  | Other | 0.00 (± 0.02) | 0.01 (± 0.55) | 0.03 |
|  |  | Decapoda | Galatheidae | *Pleuroncodes monodon* | *Pleuroncodes monodon* | 1.66 (± 12.36) | 1.74 (± 12.47) | 2.29 |
|  |  |  | Euphausiidae |  | Euphausiidae | 6.44 (± 23.63) | 7.75 (± 26.25) | 8.26 |
|  |  |  | Zoea larvae |  | Other | 0.08 (± 2.42) | 0.09 (± 2.60 | 0.17 |
|  |  |  | Decapods n/i |  | Other | 0.03 (± 1.66) | 0.06 (± 1.48) | 0.25 |
|  |  | Stomatopoda | Squillidae | *Squilla panamensis* | Other | 0.14 (± 3.61) | 0.24 (± 4.27) | 0.41 |
|  |  |  | Stomatopods n/i |  | Other | 0.05 (± 1.87) | 0.09 (± 2.33) | 0.19 |
| Teleosteii | Actinopterygii | Stomiiformes | Phosichthyidae | *Vinciguerria lucetia* | *Vinciguerria lucetia* | 19.68 (± 37.64) | 24.43 (± 37.64) | 35.90 |
|  |  | Osmeriformes | Bathylagidae | *Leuroglossus* sp. | Teleosteii | 0.26 (± 4.39) | 0.48 (± 4.72) | 1.91 |
|  |  |  | Argentinidae | *Argentina* sp. | Teleosteii | 0.00 (± 0.00) | 0.00 (± 0.21) | 0.03 |
|  |  | Myctophiformes | Myctophidae | *Benthosema panamense* | Other Myctophidae | 0.00 (± 0.00) | 0.01 (± 0.83) | 0.03 |
|  |  |  |  | *Diogenichthys laternatus* | Other Myctophidae | 2.50 (± 14.25) | 3.62 (± 15.78) | 7.79 |
|  |  |  |  | *Lampanyctus* sp. | *Lampanyctus* sp. | 4.58 (± 19.57) | 5.13 (± 17.76) | 13.57 |
|  |  |  |  | *Myctophum aurolaternatum* | *Myctophum* spp. | 1.40 (± 11.07) | 1.01 (± 7.94) | 2.76 |
|  |  |  |  | *Myctophum nitidulum* | *Myctophum* spp. | 1.21 (± 10.20) | 1.25 (± 9.49) | 2.87 |
|  |  |  |  | *Myctophum* sp. | *Myctophum* spp. | 1.00 (± 9.25) | 1.26 (± 9.10) | 3.40 |
|  |  |  |  | Myctophids n/i | Other Myctophidae | 4.47 (± 19.57) | 6.06 (± 19.75) | 14.10 |
|  |  | Perciformes | Sphyraenidae | *Sphyraena* spp. | Teleosteii | 0.70 (± 8.04) | 0.52 (± 5.50) | 1.60 |
|  |  |  | Nomeidae | *Cubiceps* spp. | Teleosteii | 1.85 (± 12.60) | 1.28 (± 9.55) | 2.76 |
|  |  |  |  | *Psenes sio* | Teleosteii | 0.30 (± 5.31) | 0.34 (± 5.00) | 0.75 |
|  |  |  | Carangidae | *Trachurus murphyi* | Teleosteii | 0.22 (± 4.43) | 0.15 (± 3.40) | 0.28 |
|  |  |  | Scombridae | Scombrids n/i | Teleosteii | 0.03 (± 1.66) | 0.01 (± 0.83) | 0.03 |
|  |  | Gadiformes | Moridae | *Physiculus* spp. | Teleosteii | 0.03 (± 1.62) | 0.00 (± 0.22) | 0.03 |
|  |  |  | Macrouridae | Macrourids n/i | Teleosteii | 0.15 (± 3.70) | 0.05 (± 1.60) | 0.19 |
|  |  |  | Merlucciidae | *Merluccius gayi peruanus* | Teleosteii | 0.14 (± 3.59) | 0.07 (± 2.19) | 0.19 |
|  |  | Clupeiformes | Engraulidae | *Engraulis ringens* | Engraulidae | 1.91 (± 12.95) | 1.39 (± 10.17) | 2.63 |
|  |  |  |  | Engraulids n/i | Engraulidae | 0.76 (± 8.29) | 0.69 (± 7.02) | 1.35 |
|  |  | Pleuronectiformes | Cynoglossidae | Cynoglossids n/i | Teleosteii | 0.05 (± 1.95) | 0.02 (± 0.88) | 0.11 |
|  |  | Syngnathiformes | Syngnathidae | *Hippocampus* sp. | Teleosteii | 0.00 (± 0.07) | 0.00 (± 0.24) | 0.03 |
|  |  |  |  | Syngnathids n/i | Teleosteii | 0.09 (± 2.85) | 0.08 (± 2.63) | 0.11 |
|  |  | Beloniformes | Exocoetidae | *Exocoetus* spp. | Teleosteii | 0.02 (± 1.32) | 0.02 (± 1.43) | 0.03 |
|  | Teleosteii n/i |  |  |  | Teleosteii | 8.91 (± 27.40) | 8.73 (± 25.52) | 15.28 |
|  | Eggs of Teleosteii n/i |  |  |  | Teleosteii | 0.00 (± 0.15) | 0.03 (± 1.66) | 0.03 |
| Urochordata |  |  | Salpidae |  |  | 0.00 (± 0.03) | 0.00 (± 0.01) | 0.03 |
| Algae |  |  | Laminariaceae |  |  | 0.03 (± 1.48) | 0.01 (± 0.53) | 0.06 |
