# Supplementary material for: Comprehensive Model of Jumbo Squid Dosidicus gigas Trophic Ecology in the Northern Humboldt Current System
Source: PLoS One. 2014 Jan 20;9(1):e85919. doi: 10.1371/journal.pone.0085919 (PMC3896428; doi:10.1371/journal.pone.0085919)
Supplement: Table S2 — Yearly description of the 55 prey taxa observed in jumbo squid stomach sampled off Peru during 2004–2011. Proportion by weight: %W; proportion by number: %N; frequency of occurrence: %O. (DOCX) [file pone.0085919.s004.docx]

Table S2. Yearly description of the 55 prey taxa observed in jumbo squid stomach sampled off Peru during 2004 - 2011. Proportion by weight: %W; proportion by number: %N; frequency of occurrence: %O.

|  |  |  |  |  | **2004** | | | **2005** | | | **2006** | | | **2007** | | | **2008** | | | **2009** | | | **2010** | | | **2011** | | |
| --- | --- | --- | --- | --- | --- | --- | --- | --- | --- | --- | --- | --- | --- | --- | --- | --- | --- | --- | --- | --- | --- | --- | --- | --- | --- | --- | --- | --- |
| **Phylum** | **Class** | **Order** | **Family** | **Species** | **%W** | **%N** | **%O** | **%W** | **%N** | **%O** | **%W** | **%N** | **%O** | **%W** | **%N** | **%O** | **%W** | **%N** | **%O** | **%W** | **%N** | **%O** | **%W** | **%N** | **%O** | **%W** | **%N** | **%O** |
| Mollusca | Gastropoda | Thecosomata | Cavoliniidae | *Cavolinia uncinata* | 0.00 | 0.00 | 0.00 | 0.85 | 1.41 | 4.02 | 0.12 | 0.76 | 1.46 | 0.00 | 0.00 | 0.00 | 0.00 | 0.00 | 0.00 | 0.00 | 0.00 | 0.00 | 0.00 | 0.00 | 0.00 | 0.00 | 0.00 | 0.00 |
|  |  |  |  | *Diacria* spp. | 4.16 | 8.71 | 13.46 | 0.00 | 0.00 | 0.00 | 0.34 | 0.89 | 1.25 | 0.43 | 0.73 | 0.84 | 0.24 | 0.47 | 2.21 | 1.01 | 2.40 | 4.46 | 0.64 | 1.33 | 3.63 | 0.25 | 2.22 | 6.55 |
|  |  |  | Thecosomata n/i |  | 0.00 | 0.00 | 0.00 | 0.00 | 0.00 | 0.00 | 0.00 | 0.19 | 0.63 | 0.00 | 0.00 | 0.00 | 0.00 | 0.00 | 0.00 | 0.00 | 0.00 | 0.00 | 0.00 | 0.00 | 0.00 | 0.00 | 0.00 | 0.00 |
|  |  | Littorinimorpha | Atlantidae | *Atlanta* spp. | 0.01 | 0.52 | 5.00 | 0.00 | 0.00 | 0.00 | 0.00 | 0.10 | 1.25 | 0.00 | 0.11 | 0.84 | 0.00 | 0.00 | 0.00 | 0.00 | 0.05 | 0.27 | 0.00 | 0.02 | 0.38 | 0.00 | 0.01 | 0.28 |
|  |  |  | Naticidae | *Natica* spp. | 0.10 | 0.51 | 2.88 | 0.00 | 0.24 | 1.34 | 0.00 | 0.32 | 2.71 | 0.00 | 0.05 | 0.84 | 0.00 | 0.05 | 0.92 | 0.00 | 0.14 | 1.49 | 0.00 | 0.02 | 0.19 | 0.00 | 0.04 | 0.57 |
|  |  | Gastropoda n/i |  |  | 0.00 | 0.03 | 0.19 | 0.00 | 0.00 | 0.00 | 0.00 | 0.01 | 0.21 | 0.00 | 0.00 | 0.00 | 0.00 | 0.00 | 0.00 | 0.00 | 0.00 | 0.00 | 0.01 | 0.18 | 0.96 | 0.00 | 0.00 | 0.00 |
|  | Bivalvia | Solemyoida larvae |  |  | 0.00 | 0.03 | 0.38 | 0.00 | 0.00 | 0.00 | 0.00 | 0.00 | 0.00 | 0.00 | 0.00 | 0.00 | 0.00 | 0.00 | 0.00 | 0.00 | 0.02 | 0.41 | 0.00 | 0.00 | 0.00 | 0.00 | 0.04 | 0.28 |
|  | Cephalopoda | Octopoda | Argonautidae | *Argonauta* sp. | 1.75 | 1.49 | 2.69 | 4.61 | 2.24 | 7.59 | 0.00 | 0.00 | 0.00 | 0.00 | 0.00 | 0.00 | 0.37 | 0.36 | 0.74 | 2.27 | 1.95 | 3.92 | 0.65 | 0.82 | 2.49 | 0.59 | 1.36 | 5.41 |
|  |  | Myopsida | Loliginidae |  | 1.43 | 1.41 | 1.54 | 0.10 | 0.11 | 0.45 | 0.00 | 0.00 | 0.00 | 0.00 | 0.00 | 0.00 | 0.00 | 0.00 | 0.00 | 0.00 | 0.00 | 0.00 | 0.00 | 0.00 | 0.00 | 0.00 | 0.00 | 0.00 |
|  |  | Oegopsida | Enoploteuthidae | *Abraliopsis affinis* | 0.00 | 0.00 | 0.00 | 0.00 | 0.00 | 0.00 | 0.00 | 0.00 | 0.00 | 0.00 | 0.00 | 0.00 | 1.17 | 0.98 | 3.87 | 5.59 | 4.58 | 10.27 | 11.15 | 9.60 | 17.97 | 7.44 | 7.40 | 13.96 |
|  |  |  | Ommastrephidae | *Dosidicus gigas* | 10.67 | 4.22 | 15.96 | 11.13 | 3.79 | 14.73 | 7.60 | 2.75 | 11.48 | 22.68 | 8.53 | 31.80 | 5.84 | 2.22 | 11.07 | 7.48 | 3.65 | 11.89 | 8.02 | 2.93 | 12.05 | 2.87 | 1.39 | 5.70 |
|  |  | Paralarve of Cephalopoda |  |  | 0.00 | 0.00 | 0.00 | 0.00 | 0.00 | 0.00 | 0.00 | 0.00 | 0.00 | 0.00 | 0.00 | 0.00 | 0.00 | 0.00 | 0.00 | 0.00 | 0.00 | 0.00 | 0.00 | 0.00 | 0.00 | 0.00 | 0.02 | 0.28 |
|  |  | Eggs of Cephalopoda |  |  | 0.00 | 0.00 | 0.00 | 0.00 | 0.00 | 0.00 | 0.00 | 0.00 | 0.00 | 0.00 | 0.00 | 0.00 | 0.00 | 0.00 | 0.00 | 0.00 | 0.00 | 0.00 | 0.00 | 0.00 | 0.00 | 0.28 | 0.28 | 0.28 |
|  |  | Cephalopods n/i |  |  | 20.06 | 16.38 | 35.58 | 16.09 | 11.88 | 29.46 | 24.98 | 21.90 | 35.49 | 19.47 | 17.81 | 30.13 | 21.97 | 19.26 | 29.34 | 33.33 | 27.42 | 40.54 | 29.06 | 25.26 | 34.61 | 34.31 | 27.18 | 45.58 |
| Arthropoda | Crustacea n/i |  |  |  | 0.79 | 1.03 | 1.92 | 0.45 | 0.52 | 1.34 | 0.00 | 0.00 | 0.00 | 0.00 | 0.00 | 0.00 | 0.26 | 0.45 | 0.74 | 0.40 | 0.34 | 0.41 | 0.71 | 0.82 | 1.15 | 0.00 | 0.00 | 0.00 |
|  | Maxillopoda | Calanoida | Aetididae | *Aetideus* sp. | 0.00 | 0.00 | 0.00 | 0.00 | 0.00 | 0.00 | 0.00 | 0.00 | 0.00 | 0.00 | 0.00 | 0.00 | 0.00 | 0.00 | 0.00 | 0.00 | 0.00 | 0.00 | 0.00 | 0.02 | 0.19 | 0.00 | 0.00 | 0.00 |
|  |  |  | Oncaeidae | *Oncaea* sp. | 0.00 | 0.00 | 0.00 | 0.00 | 0.00 | 0.00 | 0.00 | 0.00 | 0.00 | 0.00 | 0.00 | 0.00 | 0.00 | 0.00 | 0.00 | 0.00 | 0.00 | 0.00 | 0.00 | 0.16 | 0.76 | 0.00 | 0.00 | 0.00 |
|  |  |  | Calanoida n/i |  | 0.00 | 0.00 | 0.00 | 0.01 | 0.27 | 0.45 | 0.00 | 0.00 | 0.00 | 0.00 | 0.00 | 0.00 | 0.00 | 0.00 | 0.00 | 0.00 | 0.00 | 0.00 | 0.00 | 0.00 | 0.00 | 0.00 | 0.00 | 0.00 |
|  | Ostracoda |  |  |  | 0.00 | 0.00 | 0.00 | 0.00 | 0.00 | 0.00 | 0.00 | 0.00 | 0.00 | 0.00 | 0.00 | 0.00 | 0.00 | 0.00 | 0.00 | 0.00 | 0.00 | 0.00 | 0.05 | 0.18 | 0.38 | 0.00 | 0.01 | 0.28 |
|  | Malacostraca | Amphipoda | Gammaridea |  | 0.19 | 0.35 | 0.58 | 0.00 | 0.00 | 0.00 | 0.00 | 0.00 | 0.00 | 0.00 | 0.00 | 0.00 | 0.18 | 0.18 | 0.18 | 0.00 | 0.00 | 0.00 | 0.00 | 0.00 | 0.00 | 0.00 | 0.00 | 0.00 |
|  |  |  | Amphipoda n/i |  | 0.00 | 0.00 | 0.00 | 0.00 | 0.00 | 0.00 | 0.00 | 0.00 | 0.00 | 0.00 | 0.00 | 0.00 | 0.00 | 0.00 | 0.00 | 0.00 | 0.00 | 0.00 | 0.00 | 0.00 | 0.00 | 0.00 | 0.09 | 0.28 |
|  |  | Decapoda | Galatheidae | *Pleuroncodes monodon* | 0.01 | 0.10 | 0.38 | 3.58 | 3.77 | 4.02 | 0.00 | 0.05 | 0.21 | 0.00 | 0.00 | 0.00 | 3.80 | 3.50 | 4.98 | 1.07 | 1.30 | 1.76 | 1.92 | 2.25 | 2.68 | 3.81 | 3.78 | 4.84 |
|  |  |  | Euphausiidae |  | 10.49 | 12.71 | 13.27 | 2.51 | 4.29 | 4.46 | 7.64 | 8.76 | 9.19 | 5.04 | 6.10 | 6.28 | 9.32 | 11.14 | 12.18 | 6.03 | 7.10 | 7.70 | 4.14 | 4.95 | 5.16 | 2.14 | 2.71 | 3.13 |
|  |  |  | Zoea larvae |  | 0.00 | 0.00 | 0.00 | 0.15 | 0.33 | 0.89 | 0.51 | 0.53 | 0.84 | 0.00 | 0.00 | 0.00 | 0.00 | 0.00 | 0.00 | 0.00 | 0.00 | 0.00 | 0.00 | 0.00 | 0.00 | 0.00 | 0.00 | 0.00 |
|  |  |  | Decapods n/i |  | 0.00 | 0.00 | 0.00 | 0.00 | 0.00 | 0.00 | 0.00 | 0.00 | 0.00 | 0.00 | 0.00 | 0.00 | 0.00 | 0.00 | 0.00 | 0.14 | 0.31 | 1.22 | 0.00 | 0.00 | 0.00 | 0.00 | 0.00 | 0.00 |
|  |  | Stomatopoda | Squillidae | *Squilla panamensis* | 0.00 | 0.00 | 0.00 | 0.00 | 0.00 | 0.00 | 0.00 | 0.00 | 0.00 | 0.00 | 0.00 | 0.00 | 0.00 | 0.00 | 0.00 | 0.69 | 1.19 | 2.03 | 0.00 | 0.00 | 0.00 | 0.00 | 0.00 | 0.00 |
|  |  |  | Stomatopods n/i |  | 0.02 | 0.07 | 0.38 | 0.00 | 0.22 | 0.45 | 0.00 | 0.00 | 0.00 | 0.00 | 0.00 | 0.00 | 0.01 | 0.15 | 0.37 | 0.20 | 0.20 | 0.27 | 0.00 | 0.00 | 0.00 | 0.00 | 0.00 | 0.00 |
| Teleosteii | Actinopterygii | Stomiiformes | Phosichthyidae | *Vinciguerria lucetia* | 19.16 | 20.32 | 35.58 | 18.92 | 23.50 | 46.88 | 27.82 | 31.95 | 44.05 | 14.84 | 21.93 | 31.38 | 27.61 | 31.22 | 41.88 | 13.57 | 17.88 | 26.49 | 17.70 | 26.40 | 35.18 | 16.74 | 22.95 | 33.05 |
|  |  | Osmeriformes | Bathylagidae | *Leuroglossus* sp. | 0.19 | 0.14 | 0.38 | 0.43 | 0.24 | 0.89 | 0.02 | 0.24 | 1.67 | 0.69 | 0.85 | 2.09 | 0.24 | 0.70 | 3.69 | 0.34 | 0.46 | 1.89 | 0.10 | 0.54 | 1.53 | 0.34 | 0.78 | 2.85 |
|  |  |  | Argentinidae | *Argentina* sp. | 0.00 | 0.00 | 0.00 | 0.00 | 0.00 | 0.00 | 0.00 | 0.00 | 0.00 | 0.00 | 0.00 | 0.00 | 0.00 | 0.00 | 0.00 | 0.00 | 0.00 | 0.00 | 0.00 | 0.02 | 0.19 | 0.00 | 0.00 | 0.00 |
|  |  | Myctophiformes | Myctophidae | *Benthosema panamense* | 0.00 | 0.00 | 0.00 | 0.00 | 0.00 | 0.00 | 0.00 | 0.00 | 0.00 | 0.00 | 0.00 | 0.00 | 0.00 | 0.00 | 0.00 | 0.00 | 0.00 | 0.00 | 0.00 | 0.10 | 0.19 | 0.00 | 0.00 | 0.00 |
|  |  |  |  | *Diogenichthys laternatus* | 0.00 | 0.00 | 0.00 | 0.00 | 0.00 | 0.00 | 0.00 | 0.00 | 0.00 | 0.00 | 0.00 | 0.00 | 4.50 | 6.59 | 15.31 | 4.17 | 5.65 | 9.86 | 4.29 | 6.10 | 12.81 | 3.60 | 6.13 | 16.81 |
|  |  |  |  | *Lampanyctus* sp. | 2.13 | 3.50 | 11.92 | 6.25 | 7.62 | 25.00 | 8.06 | 8.06 | 20.88 | 8.60 | 11.12 | 18.41 | 3.85 | 3.73 | 13.10 | 5.10 | 5.58 | 12.16 | 3.36 | 3.32 | 8.03 | 1.47 | 1.84 | 7.41 |
|  |  |  |  | *Myctophum aurolaternatum* | 0.19 | 0.23 | 2.12 | 0.75 | 0.81 | 1.34 | 0.01 | 0.10 | 0.21 | 0.00 | 0.00 | 0.00 | 0.00 | 0.05 | 0.74 | 0.89 | 0.73 | 1.76 | 3.70 | 2.02 | 5.74 | 6.24 | 4.83 | 10.83 |
|  |  |  |  | *Myctophum nitidulum* | 0.16 | 0.17 | 0.96 | 0.94 | 1.92 | 4.91 | 2.43 | 2.18 | 4.38 | 4.22 | 4.94 | 5.86 | 0.91 | 0.94 | 2.58 | 0.23 | 0.38 | 1.08 | 2.04 | 1.65 | 4.59 | 0.53 | 0.36 | 1.99 |
|  |  |  |  | *Myctophum* sp. | 3.31 | 4.36 | 13.27 | 0.86 | 2.07 | 7.59 | 1.61 | 1.94 | 3.34 | 0.00 | 0.17 | 0.42 | 0.00 | 0.00 | 0.00 | 0.27 | 0.28 | 0.54 | 0.00 | 0.00 | 0.00 | 2.07 | 1.83 | 4.56 |
|  |  |  |  | Myctophids n/i | 4.83 | 6.82 | 20.77 | 6.34 | 9.92 | 25.45 | 4.63 | 6.31 | 21.29 | 9.40 | 13.58 | 23.01 | 2.30 | 3.02 | 7.38 | 6.16 | 7.61 | 12.43 | 2.34 | 2.93 | 7.46 | 2.16 | 3.10 | 4.84 |
|  |  | Perciformes | Sphyraenidae | *Sphyraena* spp. | 0.35 | 0.41 | 1.92 | 1.27 | 0.76 | 3.57 | 0.74 | 0.48 | 1.67 | 0.42 | 0.47 | 1.67 | 0.74 | 0.55 | 0.92 | 0.40 | 0.20 | 0.41 | 0.95 | 0.71 | 2.29 | 1.19 | 0.92 | 2.28 |
|  |  |  | Nomeidae | *Cubiceps* spp. | 0.00 | 0.00 | 0.00 | 1.82 | 0.54 | 2.23 | 1.35 | 0.49 | 2.51 | 1.62 | 1.96 | 4.18 | 2.17 | 1.71 | 2.77 | 1.18 | 0.78 | 1.62 | 1.47 | 1.23 | 3.25 | 6.94 | 4.76 | 8.26 |
|  |  |  |  | *Psenes sio* | 0.00 | 0.00 | 0.00 | 0.00 | 0.00 | 0.00 | 0.00 | 0.00 | 0.00 | 0.00 | 0.00 | 0.00 | 0.00 | 0.00 | 0.00 | 0.73 | 0.81 | 2.16 | 0.63 | 0.80 | 1.72 | 0.57 | 0.57 | 0.57 |
|  |  |  | Carangidae | *Trachurus murphyi* | 0.00 | 0.00 | 0.00 | 0.00 | 0.00 | 0.00 | 0.94 | 0.63 | 1.04 | 0.00 | 0.00 | 0.00 | 0.61 | 0.48 | 0.92 | 0.00 | 0.00 | 0.00 | 0.00 | 0.00 | 0.00 | 0.00 | 0.00 | 0.00 |
|  |  |  | Scombridae | Scombrids n/i | 0.00 | 0.00 | 0.00 | 0.00 | 0.00 | 0.00 | 0.00 | 0.00 | 0.00 | 0.00 | 0.00 | 0.00 | 0.00 | 0.00 | 0.00 | 0.14 | 0.07 | 0.14 | 0.00 | 0.00 | 0.00 | 0.00 | 0.00 | 0.00 |
|  |  | Gadiformes | Moridae | *Physiculus* spp. | 0.00 | 0.00 | 0.00 | 0.00 | 0.00 | 0.00 | 0.00 | 0.00 | 0.00 | 0.00 | 0.00 | 0.00 | 0.00 | 0.00 | 0.00 | 0.13 | 0.02 | 0.14 | 0.00 | 0.00 | 0.00 | 0.00 | 0.00 | 0.00 |
|  |  |  | Macrouridae | Macrourids n/i | 0.00 | 0.00 | 0.00 | 0.00 | 0.00 | 0.00 | 0.00 | 0.00 | 0.00 | 0.00 | 0.00 | 0.00 | 0.00 | 0.00 | 0.00 | 0.00 | 0.00 | 0.00 | 1.03 | 0.37 | 1.34 | 0.00 | 0.00 | 0.00 |
|  |  |  | Merlucciidae | *Merluccius gayi peruanus* | 0.27 | 0.29 | 0.38 | 0.00 | 0.00 | 0.00 | 0.21 | 0.01 | 0.21 | 0.00 | 0.00 | 0.00 | 0.00 | 0.00 | 0.00 | 0.00 | 0.00 | 0.00 | 0.52 | 0.21 | 0.76 | 0.00 | 0.00 | 0.00 |
|  |  | Clupeiformes | Engraulidae | *Engraulis ringens* | 0.69 | 0.34 | 1.54 | 3.70 | 2.36 | 6.70 | 0.41 | 0.26 | 0.42 | 5.19 | 4.07 | 6.69 | 5.11 | 3.64 | 6.46 | 1.53 | 1.29 | 2.03 | 0.19 | 0.10 | 0.19 | 0.80 | 0.66 | 0.85 |
|  |  |  |  | Engraulids n/i | 0.67 | 0.67 | 1.92 | 4.38 | 3.65 | 5.36 | 0.77 | 0.66 | 1.25 | 0.75 | 0.84 | 1.26 | 0.74 | 0.72 | 1.11 | 0.35 | 0.35 | 1.08 | 0.20 | 0.11 | 0.57 | 0.28 | 0.28 | 0.28 |
|  |  | Pleuronectiformes | Cynoglossidae | Cynoglossids n/i | 0.00 | 0.00 | 0.00 | 0.00 | 0.00 | 0.00 | 0.00 | 0.00 | 0.00 | 0.00 | 0.00 | 0.00 | 0.00 | 0.00 | 0.00 | 0.00 | 0.00 | 0.00 | 0.00 | 0.00 | 0.00 | 0.55 | 0.22 | 1.14 |
|  |  | Syngnathiformes | Syngnathidae | *Hippocampus* sp. | 0.00 | 0.00 | 0.00 | 0.00 | 0.00 | 0.00 | 0.00 | 0.00 | 0.00 | 0.00 | 0.00 | 0.00 | 0.00 | 0.00 | 0.00 | 0.00 | 0.00 | 0.00 | 0.01 | 0.03 | 0.19 | 0.00 | 0.00 | 0.00 |
|  |  |  |  | Syngnathids n/i | 0.00 | 0.00 | 0.00 | 0.00 | 0.00 | 0.00 | 0.00 | 0.00 | 0.00 | 0.00 | 0.00 | 0.00 | 0.00 | 0.00 | 0.00 | 0.00 | 0.00 | 0.00 | 0.62 | 0.52 | 0.76 | 0.00 | 0.00 | 0.00 |
|  |  | Beloniformes | Exocoetidae | *Exocoetus* spp. | 0.15 | 0.16 | 0.19 | 0.00 | 0.00 | 0.00 | 0.00 | 0.00 | 0.00 | 0.00 | 0.00 | 0.00 | 0.00 | 0.00 | 0.00 | 0.00 | 0.00 | 0.00 | 0.00 | 0.00 | 0.00 | 0.00 | 0.00 | 0.00 |
|  | Teleosteii n/i |  |  |  | 18.20 | 15.06 | 28.27 | 14.85 | 17.55 | 33.93 | 9.74 | 10.39 | 15.66 | 6.61 | 6.34 | 10.88 | 7.91 | 7.83 | 13.47 | 6.59 | 7.25 | 12.03 | 4.51 | 4.31 | 6.31 | 4.82 | 4.20 | 9.69 |
|  | Eggs of Teleosteii n/i |  |  |  | 0.00 | 0.00 | 0.00 | 0.00 | 0.00 | 0.00 | 0.00 | 0.00 | 0.00 | 0.04 | 0.42 | 0.42 | 0.00 | 0.00 | 0.00 | 0.00 | 0.00 | 0.00 | 0.00 | 0.00 | 0.00 | 0.00 | 0.00 | 0.00 |
| Urochordata |  |  | Salpidae |  | 0.00 | 0.00 | 0.00 | 0.00 | 0.00 | 0.00 | 0.00 | 0.00 | 0.00 | 0.00 | 0.00 | 0.00 | 0.00 | 0.00 | 0.00 | 0.00 | 0.00 | 0.14 | 0.00 | 0.00 | 0.00 | 0.00 | 0.00 | 0.00 |
| Algae |  |  | Laminariaceae |  | 0.00 | 0.00 | 0.00 | 0.00 | 0.00 | 0.00 | 0.06 | 0.05 | 0.21 | 0.00 | 0.00 | 0.00 | 0.16 | 0.04 | 0.18 | 0.00 | 0.00 | 0.00 | 0.00 | 0.00 | 0.00 | 0.00 | 0.00 | 0.00 |
