# Supplementary material for: Comprehensive Model of Jumbo Squid Dosidicus gigas Trophic Ecology in the Northern Humboldt Current System
Source: PLoS One. 2014 Jan 20;9(1):e85919. doi: 10.1371/journal.pone.0085919 (PMC3896428; doi:10.1371/journal.pone.0085919)
Supplement: Table S3 — Result of the Kruskal-Wallis test performed on the 11 dietary groups according to Size, Distance to the shelf-break, Season and SSTA. Significant differences are in bold. (DOCX) [file pone.0085919.s005.docx]

Table S3. Result of the Kruskal-Wallis test performed on the 11 dietary groups according to Size, Distance to the shelf-break, Season and SSTA. Significant differences are in bold.

|  | **Size (df=3)** | | **Distance to the shelf (df=3)** | | **Season (df=3)** | | **SSTA (df=3)** | |
| --- | --- | --- | --- | --- | --- | --- | --- | --- |
| **Dietary group** | **H** | **p-value** | **H** | **p-value** | **H** | **p-value** | **H** | **p-value** |
| *Dosidicus gigas* | 55.78 | **<0.001** | 2.78 | 0.42 | 3.31 | 0.35 | 30.21 | **<0.001** |
| Others Cephalopoda | 66.12 | **<0.001** | 12.92 | **0.01** | 21.47 | **<0.001** | 28.52 | **<0.001** |
| Euphausiidae | 73.26 | **<0.001** | 8.69 | **0.03** | 45.86 | **<0.001** | 75.1 | **<0.001** |
| *Pleuroncodes monodon* | 4.73 | 0.19 | 10.67 | **0.01** | 7.47 | 0.06 | 44.79 | **<0.001** |
| *Vinciguerria lucetia* | 140.42 | **<0.001** | 46.09 | **<0.001** | 163.19 | **<0.001** | 78.52 | **<0.001** |
| *Lampanyctus* sp. | 41.16 | **<0.001** | 4.92 | 0.18 | 37.78 | **<0.001** | 18.41 | **<0.001** |
| Myctophidae | 32.61 | **<0.001** | 11.09 | **0.01** | 36.55 | **<0.001** | 24.99 | **<0.001** |
| *Myctophum* spp. | 57.89 | **<0.001** | 34.09 | **<0.001** | 31.78 | **<0.001** | 43.97 | **<0.001** |
| Engraulidae | 4.56 | 0.21 | 29.89 | **<0.001** | 37.62 | **<0.001** | 15.92 | **<0.001** |
| Teleosteii | 6.77 | 0.08 | 7.01 | 0.07 | 5.33 | **p<0.001** | 2.57 | 0.46 |
